# Acknowledgment to the Reviewers of *Tropical Medicine and Infectious Disease* in 2022

**DOI:** 10.3390/tropicalmed8020070

**Published:** 2023-01-18

**Authors:** TropicalMed Editorial Office

**Affiliations:** MDPI AG, St. Alban-Anlage 66, 4052 Basel, Switzerland

High-quality academic publishing is built on rigorous peer review. *TropicalMed* was able to uphold its high standards for published papers due to the outstanding efforts of our reviewers. Thanks to the efforts of our reviewers in 2022, the median time to first decision was 16 days and the median time to publication was 39.5 days. Regardless of whether the articles they examined were ultimately published, the editors would like to express their appreciation and thank the following reviewers for the time and dedication that they have shown *TropicalMed*:
A. N. AnoopkumarKenta TeruyaAbdiel Martín-ParkKentaro YamadaAbdulkarim Jafar KarimKerry RedicanAbhinav SinhaKevin FonsecaAdama DialloKlaus H. HoffmannAdi BeharKlaus HeunerAdriaan VosKoert RitmeijerAdrian OoKonstantin SharovAdriana AnitaKonstantinos ArsenopoulosAdriana SilvaKorawan SringarmAdy WirawanKovy Arteaga-LiviasAgieshkumar Balakrishna PillaiKrishna C. ChintaAgustin Lugo-RadilloKrishnaswamy Gopalan TirumurugaanAhmad Ali Hanafi-BojdKrutika MediwalaAhmet BaydinKun LiAishah H. AzilKun SongAjay Kumar VermaKun YangAkram Hernández-VásquezKwabena Frimpong Manso OpuniAlberto Comesaña-CamposLaibané Dieudonné DahourouAlberto MacchiLakshmi Prasad G.Albie De FreyLarisa ProzorovaAleš ChrdleLaura LuccheseAlessandro SapienzaLei HuangAlex MammenLeo Kei IwaiAlexander AlekseevLeonardo AugustoAlexandr Anatoljevich StekolnikovLeonardo BascoAlexandra EhrensLeonardo Catalano IniestaAlexandra GambaryanLeonardo KoerichAlexandra StewartLeonardo Mariño-RamírezAlexandru Bogdan CiubaraLeonelo BautistaAlfred T. HardingLianet MonzoteAli GoliLíbia Zé-ZéAlicia Harvey-VeraLida FanAlireza MohammadiLilia GutierrezAlsaid Ahmed AlmetwallyLilian SosaAltaira D. DearbornLilian Spencer-ValeroAly Salimo MuadicaLina TomasoniAlyssa B. EvansLindsey ShieldsAmalia AnastasopoulouLisiane Morelia Weide AcostaAman Ullah KhanLi-Yen ChangAmar Nath ChatterjeeLizette KoekemoerAmit SinghLong ChenAna MontoyaLong Thanh PhamAna ReisLongxiang LiAna SanchezLoredana Sabina Cornelia ManolescuAna VasićLorenzo DomenisAnak IamaroonLouie Mar A. GangcuangcoAnanya Tupaki-SreepurnaLourdes LledoAnders BjörkmanLua Perimal-LewisAndré Conceição PereiraLuca FreddiAndré Lima AiresLuciana ReisAndréa Alice Da SilvaLuciana Silva-FlanneryAndrea BertkeLucie PaloqueAndrea CioffiLuigi Segagni LusignaniAndrea ManfrinLuigia BruglieraAndréia Moreira Dos Santos CarmoLuis Alberto Sanchez VargasAndrew DiNardoLuis Fernando MachadoAndrew EdmondsLuis Furuya KanamoriAndrew RamsayLuis Roberto De Camargo GonçalvesAndriy SverstyukLuis ToroAndy LeisewitzLydia MosiAngela BarbosaLyle R. PetersenAngela M. Garcia SanchezMaciej FrantAngelita Cristine De MeloMaciej KochanowskiAnil V. KumarMadeeha MalikAnisuz ZamanMagdalena Nowak-ChmuraAnisuzzaman AnisuzzamanMahesh KasthuriAnkur KaushalMahmoud Abo-AshourAnna Boroń-KaczmarskaMahmoud NassarAnna MertasMahmut SafakAnnarita BottaMai Salah El Din Mohamed HelmyAntonio Guerrero EspejoMaja MiskulinAntonio Olivas-MartinezMajed H. WakidAnup BastolaMan Lai CheungAnwar A. SayedManas KotepuiAparna KrishnavajhalaMarcel S. MirandaApichat VittaMárcio Poletti LauriniAravinthkumar JayabalanMarcos Leoni GazariniArchana MishraMarek AsmanAriane De Jesus Sousa-BatistaMargaret BakerAriane DorMargarida DuarteArmando BaenaMaria Adela ValeroArmando Silva-AlmodóvarMaria C. CarrasquillaArnau Casanovas-MassanaMaria Gabriella VersoAroonlug LulitanondMaria Isabel Nogueira Di AzevedoArtem MetlinMaria MatuzArumugam RajaveluMaria PernasArunee AhantarigMaria SzymankiewiczAshuin Kammar-GarcíaMarian Sorin PaveliuAswi AswiMariana Côrtes BoitéAtchara PhumeeMarie BorkovcovaAtef KhalilMariel Aguilar-DomínguezAthanasios AngelouMarino VilovićAthanasios SachlasMario I. OrtizAtiqa AmbreenMariola BochniarzAurélie HennebiqueMaritza Omaña-MolinaAyesha HassimMarius CoetzeeAyman El-BadryMarius MasiulisAyola AdegnikaMariusz NiemczykAzger DusthackeerMark W. RobinsonAzidah Abdul KadirMark W. SonderupAzize DemirpolatMarta ArsuagaBabak EshratiMarta Diaz-MenendezBanhi BiswasMarta G. CavalcantiBaptiste DemeyMarta Helena BranquinhaBashir Ahmad FomdaMarta RoratBasu Dev PandeyMartha Suárez MutisBayodé Roméo AdégbitèMartin LangerBeatriz BrenerMartina LaidemittBeatriz RobredoMarwa AttiaBebiana CondeMary J. KastenBehrouz Nezafat MaldonadoMarylin HidalgoBehzad KianiMateusz GrajekBela KocsisMathuros TipayamongkholgulBenjamin DavidoMatías CastellsBenjamín Valladares-CarranzaMatteo RiccòBernard A. OkechMaurizio NordioBernard J. HoetMax MaurinBernardo VegaMd. Abdus SoburBetty BalikagalaMd. Rabiul IslamBijay Ranjan MirdhaMd. Siddikur RahmanBijaya PadhiMd. Tanvir RahmanBin ZhanMegha SharmaBoris IoninMeghnath DhimalBrandon J. BeddingfieldMehdi BamorovatBrian Shyue Wen ChongMehdi NateghpourBrice AutierMehmet Tahir HuyutBusaba SupawattanabodeeMehran GhaemiCaitlin A. O’BrienMeksianis NdiiCarissa Embury-HyattMelinda HerbathCarla Maria Batista Ferreira PiresMelissa Hanzen PinnaCarlo Alberto MaroneseMeredith B. BrooksCarlos Felix MarinaMerid Negash GetahunCarlos Graeff-TeixeiraMian NavedCarlos J CarrascoMichael AlkanCarlos Mestanza-RamónMichael MacDonaldCarlos Sandoval-JaimeMichael RamharterCarmen María Sandoval PachecoMiftahuddin MiftahuddinCarmen PavelescuMihaela KavranCarmen Rojas-MartínezMing-Chih YuCarmen Soria-SegarraMiroslav FajfrCarolina Perez-HeydrichMitsuru SadaCassiano VictóriaMoeti Oriel TaioeCatalin TomaMohamed FawzyCatalina AvendañoMohamed LounisCatherine A. GordonMohamed MahdiCatherine M. BrownMohamed NasserCauê Benito ScarimMohammad Tahir SiddiquiChandika D. GamageMohammad Yasir EssarChandika GamageMojakgomo Hendrick MotswalediChandrima ChatterjeeMónica Ríos-SilvaChangzhen WangMonique Da Rocha Queiroz LimaChaofan LiMoritoshi IwagamiChe Julius NgwaMorshed NasirCheerawit RattanapanMostafa DianatinasabCheng-Chieh YenMostafa F. N. AbushahbaChenjian GuMoussa SangareChia-Po FuMuhammad FarooqChoirul AminMuhammad Nauman ZahidChoosak NithikethkulMuhammad ZeeshanChristina M. NewmanMuhammet KarakavukChristina MergenthalerMurat KirişciChristina S. ThorntonMuriel Ramírez-SantanaChristopher VitekN. Rich NguyenChuan Hun DingNaazneen MoollaChun-Yu LinNajm AlshahraniClarissa BorgesNakwon KwakClaudia CodeçoNancy Odurowah Duah-QuashieClaudia Figueiredo-MelloNaoyuki ItohClaudimar Pereira Da VeigaNarendran Pradeep KumarClemax Couto Sant’AnnaNatalia Wiktorczyk-KapischkeClemente MossoNatalya GlushkovaColleen AldousNataša KnapDan-Alexandru TocNayab BatoolDaniel H. ParisNeda Milevska KostovaDaniel TurudićNeha A. ThakreDaniel WrightNgonda SaasaDaniel YoukeeNicolae PopaDaniela ArosioNikolaos MelasDaniela RandoNilanjan LodhDaouda KassiéNina S. YanchevaDavid A. HolcombNiruwan TurnbullDavid CouvinNobumichi KobayashiDavid FidockNoemia Teixeira De Siqueira-FilhaDavid FrancoNoor Al-Huda Ali A. H. SaeedDavid KrantzNorhan Abd El-AzizDavid LarsenNorma Velázquez-GuadarramaDavid Lawrence SacksOana SandulescuDavid M. AllenOlayinka IdrisDavid RollinsonOleksii SkorokhodDavid ŠmajsOliver Chun Hung KwokDavide SoglianiOliver Mendoza-CanoDebashis DuttaOlivia DorneanuDeborah J. MillsOlivier MaillardDeborah NgOsman AktaşDeema DababsehOsvaldo LoquihaDemba SarrOtávio EspíndolaDenis KrivoguzPablo F. CuervoDesmond O. AcheampongParthasakha DasDevadas KrishnakumarPascalina Chanda-KapataDevi Shankar SumanPatricia EscandónDevojit Kumar SarmaPatricia GravesDhruvajyoti RoyPatricia Hernández-RodríguezDiana ErazoPatrick PalmieriDiawo DialloPatrick Stephan SebastianDimitri KakabadsePatrizia CaprariDimitris TsakogiannisPaul AirsDinesh MittalPedro CattanDinkar SahalPer NordinDivakar HemadriPerugu ShyamDivya NairPeter Kotsoana MontsoDmitry VerbenkoPeter LarsonDominic Selorm Yao AmuzuPeter MwabaDominika SalamonPeter MwangiDominique J. BicoutPeter Seah Keng TokDong Keon YonPeter ThompsonDongdong CaoPetr ZemanDoris Gomez-CamargoPetrick Ramesh K. PeriyasamyDorte Bek FolkvardsenPhilip El-DuahDragana ValjarevicPhilip J. BudgeDrishya DiwakerPicha SuwannahitatornDulce GomesPierre RoquesEdgar CambazaPinar KorkmazEdgar Tello-LealPiyanan TaweethavonsawatEdward StephensPornlada NuchnoiEdwin MichaelPourya GholizadehEfrén Murillo-ZamoraPrabhdeep KaurEggi ArguniPragya AgarwalaEl Mehdi LotfiPrakasha KempaiahElena BencurovaPrasanth BalasubramanianElena CiguPratima GuptaElena PomariPriscila Mayrelle Da Silva CastanhaEleni PalliPruthu ThekkurEleni PatsoulaPugazhenthan ThangarajuElif KaraaslanPuja KumariElizabeth OmukundaPuo-Hsien LeElizaveta StarodubovaPyrpasopoulou AthinaElodie Monchatre-LeroyQin SunElpidius J. RukambileQu ChengEman AbdelsameeaRafael Stuani FlorianoEman KhalifaRafaella Sayuri IoshinoEmanuel GoldmanRai Campos SilvaEmanuel L. PeterRaja DasEmanuele NicastriRajalaxmi Rajammal RamasamyEmilie JavelleRajendra MaharajEmily McDermottRakesh P. SuseelaEmmanuel NjogaRaktham MektriratEmmanuel SerranoRaman VelayudhanEnrica TorrettaRamendra Pati PandeyEnrique Méndez BolainaRamesh DhimanEric James NillesRamesh KumarErnest TamboRania TohmeErum KhanRanjan PremaratnaEsther M. EllisRaquel Fernandez-GarciaEvdoxia KyriazopoulouRasoul MirzaeiEwen C. D. ToddRatchadawan AukkanimartExequiel ScialfaRauni KivistöFabiana Volpe-ZanuttoRavindra A. PrabhuFaez Firdaus Jesse AbdullahRazique AnwerFaisal IsmailRăzvan-Ionuț PopescuFaiz Ullah KhanRebecca AppletonFakhri JeddiRegina Helena PiresFanhao NieRegina PodlasinFarhad NikkhahiRegragui TakiFatemeh TabatabaieReham M. El-TarabiliFatih HatipogluReinhold P. LinkeFátima AmaroRenata Heisler NevesFatmawati FatmawatiRenata Pimentel Bandeira De MeloFeliciano Milian SuazoRenata RetkuteFelipe RidolfiRen-Huei FuFengqing ChaoRenu WickremasingeFernanda MorgadoRichard CashFernando Chanisso MulandaneRichard GuerrantFernando JacinaviciusRichard NgandoloForoud ShahbaziRiitta A. DlodloFotinie NtzioraRini RachmawatiFrancine CournosRobert SusloFrancisco Chiaravalloti-NetoRobert SusłoFrank Badu OseiRoberta Lima CaldeiraFrank BeardRobiatul AdawiyahFrank SchurenRocío ChecaFrank W. AvilaRoderick HayFredy A. Rivera PaezRodrigo M. CorderFriederike EbnerRodrigo Rollin-PinheiroGabriel Zorello LaportaRodrigo Saar GomesGanesh YadaigiriRoman KuchtaGenevieve André-FontaineRoman LeontovyčGerald M. MkojiRonaldo Cesar Borges GryschekGerardo Santos-LópezRoy MarzoGerman PerezRuchi BhandariGhyslain Mombo-NgomaRudi CassiniGiacomo CasaliniRupak NagraikGilbert OctaviusRuzilawati Abu BakarGiovanna Barba-SpaethSaad AlghamdiGiovanni Francesco PellicanoSaber EsmaeiliGiuditta De LorenzoSabri HacıoğluGloria Ivy MensahSadia ShakeelGodwin W. NchindaSaito NobuoGonçalo SantinhaSajjad AhmadGonzalo A. ColladoSamantha StonbrakerGordana KovacevicSami SimsekGregory M. AnsteadSamson OlaniyiGriselda BerberiánSandra GavaGuanhong WangSantiago Mirazo VillarGuibehi Benjamin KoudouSantosh DhakalGurpreet SinghSara Silva PereiraGustavo CampagnaroSaravanan PalaniGustavo FontechaSaravu R. NarahariGuy W. Soo HooSaurabh RamBihariLal ShrivastavaHae Suk CheongSeok Mui WangHafiz RizwanSérgio Santos De AzevedoHagen FrickmannSerhii NazarovetsHala HusseinSevero Vázquez PrietoHalima DawoodShakir AliHamed Ghasemzadeh‐MoghaddamShareen IqbalHamid StajiShelly RobertsonHamza AvciogluSherif ElkafrawyHana RohanShigeyuki KanoHani Amir AouissiShimon HarrusHaoyi WangShoaib FreedHargobinder KaurShubhi KaushikHaroon AkbarShüné OliverHarry AcquatellaShuowei CaiHarry MoultrieShuxin ZhangHarry TagborSibel Altunişik TopluHassan MahmoudiSilva KouyoumjianHéctor Hugo GarcíaSílvia I. SardiHéctor Manuel Sánchez CastellanosSimon D. Van HarenHedwin Kitdorlang DkharSimón Pedro Izcara PalaciosHeinz-Josef SchmittSinnathamby Noble SurendranHelen FogartySitong LuoHelen StaggSivalingam NalliahHelena JoséSivapong SungpraditHelene Santos BarbosaSnehil DixitHéloïse LucaccioniSnežana MedićHenk SchalligSolai Ramatchandirane PrabagaranHenri AstenSoma ChattopadhyayHenricus Leo Bernardus Maria KlaasenSomanon BhattacharyaHillary K. KiprutoSonja RadojičićHiroshi SatoSoufien SghaierHiroshi ShimodaSrikanth UmakanthanHisham ImadSrinivas Reddy PallerlaHridesh Kumar MishraStefano DastoliHudson Alves PintoStephan SchmidHusam QanashSubhas KhajanchiHüseyin CanSudarat ChadsuthiHussain HamidahSudip DuttaI. Gede Nyoman Mindra JayaSue NangI. Gusti Ngurah Edi PutraSuilan ZhengIan Kim B. TabiosSulaiman LakohIe-Bin LianSuman WattalIgor Cavallini JohansenSuparat PhuanukoonnonIgor DumicSupawadee PirataeIjaz GulSupawat ChatchenIkechukwu AchilonuSutas SuttiprapaIlana BalassianoSuzana OtaševićIldefonso Fernández SalasSvetoslav Nanev Slavovİlknur KaleliSvjetlana RausInge M. KrijgerSweety SamalIoannis PetrakisSwetalina PradhanIrena TabainSyed Mohammed BasheeruddinIrina ChelyshevaSzu-Chieh ChenIris BiebachT. G. A. Nilmini ChandrasenaIrit NachtigallTaeyoung KongIsabela Maria Bernardes GoulartTaha BatIsabelle BonmarinTaha MehanyIskren KaftandjievTai-Ho ChenIsmail OdetokunTaiwo AremuIssa SyTakahiro AsamiIuliana NenuTakashi YoshiyamaIvan PavlovićTakehiko KobayashiIzabela RaganTakeshi HattaIzabela ZakrockaTakumi SaitoIzabella RządTamara K. DzagurovaJacek ŻmudzkiTamara Nunes Lima-CamaraJaidip JagtapTapan BhattacharyyaJaime Bustos-MartínezTassanee SilawanJaishree RamanTatsuo ShiodaJamal SarvariTawsifur RahmanJames LaCourseTerence OdochJames S. MiserTeresa RochaJames Van EttenTerry A. KleinJamille G. DombrowskiThaigarajan ParumasivamJanaína Capelli-PeixotoThiago Dias SartiJanani ThapaThiruvenkadan Aranganoor KannanJane LabadinThundon NgamprasertchaiJanine F. R. SeetahalTiantian JiangJasminka TalapkoTijana ŠtajnerJavad AlizargarTimothy A. EricksonJayanti Mania PramanikTimothy EricksonJazzmin ArrivillagaTimothy N. C. WellsJean Marcel DeguenonTimotius Ivan HariyantoJeffrey E. HarrisTing-Wu ChuangJelena S. DrulovićTiong Kai TanJeng-Wei TsaiTolstenkov OlegJeniffer CarrilloTom BenekeJennifer D. Foulke-AbelTomasz BogielJenny ShieldTomoyuki KanazawaJeongwoo LeeTorgny SunnerhagenJeremy V. CampTuba BaygarJeronimo AlencarTuija GaddJessica ElfUlrich OrdaJessica RauchUmberto MoliniJèsus Maria Perez JimenezUrsula PanznerJhon Castaño-OsorioUrszula NawrotJiahui LeiUsha Narayana PillaiJiajun ZhuUsman Ayub AwanJiayue YanUttam GhoshJin GanValentina CuriniJinlin LiValerie Le SageJinxin ZhaoVan Dinh TranJiri DreslerVan Lun LowJoan Francesc Fondevila GascónVanja TesicJoao Aristeu Da RosaVarakorn KosaisaveeJohad F. KhouryVed Prakash VermaJohn C. TrefryVeerachai ThitapakornJohn DzimianskiVeerasak PunyapornwithayaJohn Edward ThornesVenkata Nagaraj KakaraparthiJohn MackenzieVeronica R. FloresJohn P. A. LusinguVesna MilicevicJorge CervantesVicky Panduro-CorreaJose Alejandro Martinez-IbarraVictor Dahl MathiasenJosé Angelo Lauletta LindosoVictor I. BandJosé Brites-NetoVictor Manuel Sanchez-CorderoJosé Bustos-ArriagaVictoria Pando-RoblesJosé H. PilottoVijeta SharmaJose Luis Perez ArellanoVikas KushwahaJosé Roberto Machado-SilvaVinoth RajendranJosé SilvaViravarn LuviraJosé-Luis Perez-ArellanoVishal Singh NegiJoseph A. WestrichVishma Pratap SurJoseph ServadioVivornpun SanprasertJoseph W. MagonaVladimir SavranskyJosh ColstonVladimir VukovićJoshua KamaniWalter MuleyaJovana TodorovicWanessa Trindade ClementeJuan David Ospina-VillaWanida MalaJuan Luis Delgado-GallegosWatcharapong PiyaphaneeJuan Luis Muñoz BellidoWei Boon YapJudit Villar-GarcíaWei XiaoJulio Vicente Figueroa-MillánWeike ZhouJun HangWilber SabiitiJun Jie Benjamin SengWilson L. MandalaJurema Corrêa Da MotaWojciech ZygnerJyoti DasXóchitl TrujilloJyoti RanaYadollah Abolfathi MomtazKaja Murali-KrishnaYafei WangKalyan DasYe ZhangKamenna VutovaYekti WidyaningsihKamonwon IenghongYin-Chou HsuKankoé SallahYin-Ping WongKanok PreativatanyouYoshikazu Honda-OkuboKarien LabuschagneYuko HaradaKarl SeydelZachary A. YetmarKasi VegesanaZahid Ahmad ButtKatalin KoltaiZaid YounesKatarzyna Buńkowska-GawlikZary YahayaKatharina KusejkoZbigniew ArentKatherine AppletonZehra EdisKathrin ArndtsZeno BisoffiKathryn A. McFarlaneZhen WangKátia Da Silva CalabreseZia SindhuKazuki IdeZoi AthanasakopoulouKennio Ferreira-PaimZurisaday Santos-Jimenez


